# Editorial: Hemostatic Challenges in Pediatric Critical Care Medicine

**DOI:** 10.3389/fped.2021.697921

**Published:** 2021-07-12

**Authors:** Gemma L. Crighton, Oliver Karam, Marianne E. Nellis, Simon J. Stanworth

**Affiliations:** ^1^Department of Haematology, Royal Children's Hospital, Melbourne, VIC, Australia; ^2^Division of Pediatric Critical Care Medicine, Children's Hospital of Richmond at VCU Richmond, Richmond, VA, United States; ^3^Division of Pediatric Critical Care Medicine, NY Presbyterian Hospital—Weill Cornell Medicine, New York, NY, United States; ^4^NHS Blood and Transplant; Oxford University Hospitals NHS Foundation Trust; Radcliffe Department of Medicine and Oxford BRC Haematology Theme, University of Oxford, Oxford, United Kingdom

**Keywords:** pediatric, critical care, haemostasis, coagulation, bleeding, transfusion, hemorrhage

“*When one thinks about the current state of critical care for children, do we not wonder why and how we got here, how we are doing, and where we are going?”* (John J. Downes—US pediatric intensivist).

*How we got here?*

Hemostasis (derived from Greek *haimostasis*) literally means arrest of bleeding (1). Our knowledge of hemostasis has greatly advanced since ancient Greek times, when Homer described in *The Iliad* and *The Odyssey*, the treatment of war wounds with compression, bandages, cautery and styptics (2). Hemostasis is a complex and highly regulated process, involving the vascular system, platelets, coagulation factors, fibrinolytic systems, serine protease, kinin, and complement systems (3). This finely balanced system functions effectively to protect against both bleeding and thrombotic complications.

Understanding hemostatic dysfunction firstly relies on understanding what is normal. Maureen Andrews was a pioneer in the field of pediatric hemostasis when she reported on the vast differences seen between the hemostatic systems of neonates and adults and reported reference ranges for common coagulation tests (4, 5).

*How are we doing?*

Blood transfusions are one of the top five overused treatments and in many clinical settings provide no or negligible benefit and potentially expose patients to harm (6). Children are at least twice as likely as adults to have an adverse reaction secondary to transfusion (7, 8). In the Platelets for Neonatal Thrombocytopenia (PlaNeT-2) trial, platelet transfusions to support higher platelet counts in preterm neonates cause more harm than restrictive practice, giving platelets at lower thresholds (9). A key concept of pediatric patient blood management is patient and family-centered care (10). How would children and their families feel about being treated with potentially unnecessary or even harmful interventions?

In this collection of articles, Nair and Parker provide an overview of hemostasis and its regulation, discussing developmental hemostasis, laboratory tests used to evaluate hemostasis in children and their limitations. Gillespie and Doctor discuss how red blood cells (RBC)s contribute to hemostasis, from RBC biomechanics to cell-to-cell signaling and humoral influences and finally RBC's role in thrombosis and bleeding. Davenport and Sola-Visner describe the “*developmentally unique neonatal hemostatic system,”* discussing common neonatal bleeding presentations, current and emerging coagulation tests and therapeutic interventions.

In times of health, the hemostatic systems of neonates and children are considered physiologic or balanced, this balance may be greatly disrupted in the context of illness, sepsis, trauma, major surgery, liver disease, mechanical circulatory support, [e.g., extracorporeal membrane support (ECMO) or ventricular assist devices (VAD)s] and medications.

In this series, Bulut et al. describe the hemostatic balance in pediatric acute liver failure, limitations of current coagulation testing, the role of viscoelastic haemostatic testing and potential treatment strategies. Haas and Cushing review trauma-induced coagulopathy in children, discussing transfusion strategies in pediatric trauma patients and veno-thromboembolism.

Drop et al. evaluate the risk factors for hemostatic complications in pediatric ECMO patients, alternative anticoagulants and the association between coagulation tests and hemostatic complications. Ghbeis et al. review hemostasis in children with VAD and introduce ACTION (Advanced Cardiac Therapies Improving Outcomes Network), a collaborative international network aiming to improve outcomes for pediatric VAD patients.

Clinically significant bleeding is encountered in approximately one tenth of critically ill children (11, 12) and is a major cause of morbidity and mortality. Transfusions are frequently given to critically ill children to prevent or treat clinical bleeding. Aran et al. provide an overview of bleeding in critically ill children, definitions of bleeding and discuss BASIC (Bleeding Assessment Scale in Critically Ill Children), the first diagnostic criteria for describing bleeding applicable to critically ill children. Kahn et al. evaluate platelet and plasma transfusions in children, highlighting the lack of evidence to support their use and the variability in practice for transfusion indications and thresholds. Crighton and Huisman's two-part article discusses diagnostic tools to measure fibrinogen, fibrinogen replacement products and clinical indications in critically ill children.

What is striking from this collection of articles, is the lack of high-quality evidence to support hemostatic decision making in critically ill children. Only 18 RCTs were discussed amongst the 11 articles, that evaluated either transfusion support, fibrinogen supplementation or anticoagulation in different cohorts of sick neonates or children.

*Where we are going?*

Several areas recommended for future research include better understanding the coagulopathy of pediatric acute liver failure, trauma-induced coagulopathy including fibrinolysis shutdown in traumatic brain injury (13) and the role of the endothelial glycocalyx and coagulation in pediatric trauma and sepsis (14, 15). New hemostatic methods need evaluation in children specifically, thrombin generation testing and whole-blood hemostatic assays and platelet function assays in neonates. Research is needed to establish the relationship between coagulation parameters and clinical bleeding risk in children and neonates. BASIC bleeding definitions require validation in large patient cohorts and in relation to clinical outcomes.

RCTs are needed to guide recommendations for plasma and platelet transfusions in children, for prophylactic and therapeutic indications and to determine the optimal fibrinogen replacement product and triggers for fibrinogen supplementation in critically ill children in different clinical settings. Studies are also needed to evaluate newer platelet products, such as cold-stored platelets, thrombopoietin mimetics, and artificial platelets. In pediatric ECMO and VAD patients, evidence is needed to reduce the frequency of bleeding and thrombotic events and improve patient outcomes. Given the significant variability in the management of both ECMO and VAD patients it is important to define best practice and reduce treatment-related adverse events. Research priorities in pediatric trauma include determining the optimal resuscitation strategy (including the role of whole blood and fibrinogen), the effect of pre-hospital tranexamic acid and prevention of veno-thromboembolism.

In summary, there remains an urgent need for further research in critically ill neonates and children to guide the evaluation of hemostasis and bleeding risk and its optimal treatment. As seen with recent publications in adults (16, 17), it is possible to conduct high-quality RCTs in critical care settings and we should strive for the same in pediatrics. It is essential to consider the adverse effects of transfusion, limit unnecessary use and ensure effective implementation of any research findings.

## Author Contributions

All authors listed have made a substantial, direct and intellectual contribution to this research topic. GC conceptualized and wrote the first draft of this manuscript. OK, MN, and SS contributed to manuscript revision, read, and approved the submitted version.

## Conflict of Interest

The authors declare that the research was conducted in the absence of any commercial or financial relationships that could be construed as a potential conflict of interest.
